# Associations Between Substance Use and Instagram Participation to Inform Social Network–Based Screening Models: Multimodal Cross-Sectional Study

**DOI:** 10.2196/21916

**Published:** 2020-09-16

**Authors:** Brandon G Bergman, Weiyi Wu, Lisa A Marsch, Benjamin S Crosier, Timothy C DeLise, Saeed Hassanpour

**Affiliations:** 1 Recovery Research Institute Center for Addiction Medicine Massachusetts General Hospital, & Harvard Medical School Boston, MA United States; 2 Department of Biomedical Data Science Geisel School of Medicine at Dartmouth Hanover, NH United States; 3 Center for Technology and Behavioral Health Geisel School of Medicine at Dartmouth College Lebanon, NH United States; 4 Departments of Biomedical Data Science, Computer Science, and Epidemiology Geisel School of Medicine at Dartmouth Hanover, NH United States; 5 Department of Mathematics and Statistics Universite de Montreal Montreal, QC Canada

**Keywords:** substance use, social network sites, health risk, screening, machine learning, social media, Instagram, alcohol, drug

## Abstract

**Background:**

Technology-based computational strategies that leverage social network site (SNS) data to detect substance use are promising screening tools but rely on the presence of sufficient data to detect risk if it is present. A better understanding of the association between substance use and SNS participation may inform the utility of these technology-based screening tools.

**Objective:**

This paper aims to examine associations between substance use and Instagram posts and to test whether such associations differ as a function of age, gender, and race/ethnicity.

**Methods:**

Participants with an Instagram account were recruited primarily via Clickworker (N=3117). With participant permission and Instagram’s approval, participants’ Instagram photo posts were downloaded with an application program interface. Participants’ past-year substance use was measured with an adapted version of the National Institute on Drug Abuse Quick Screen. At-risk drinking was defined as at least one past-year instance having “had more than a few alcoholic drinks a day,” drug use was defined as any use of nonprescription drugs, and prescription drug use was defined as any nonmedical use of prescription medications. We used logistic regression to examine the associations between substance use and any Instagram posts and negative binomial regression to examine the associations between substance use and number of Instagram posts. We examined whether age (18-25, 26-38, 39+ years), gender, and race/ethnicity moderated associations in both logistic and negative binomial models. All differences noted were significant at the .05 level.

**Results:**

Compared with no at-risk drinking, any at-risk drinking was associated with both a higher likelihood of any Instagram posts and a higher number of posts, except among Hispanic/Latino individuals, in whom at-risk drinking was associated with a similar number of posts. Compared with no drug use, any drug use was associated with a higher likelihood of any posts but was associated with a similar number of posts. Compared with no prescription drug use, any prescription drug use was associated with a similar likelihood of any posts and was associated with a lower number of posts only among those aged 39 years and older. Of note, main effects showed that being female compared with being male and being Hispanic/Latino compared with being White were significantly associated with both a greater likelihood of any posts and a greater number of posts.

**Conclusions:**

Researchers developing computational substance use risk detection models using Instagram or other SNS data may wish to consider our findings showing that at-risk drinking and drug use were positively associated with Instagram participation, while prescription drug use was negatively associated with Instagram participation for middle- and older-aged adults. As more is learned about SNS behaviors among those who use substances, researchers may be better positioned to successfully design and interpret innovative risk detection approaches.

## Introduction

### Enhancing the Utility of Technology-Based Strategies to Detect Substance Use

More than 1 in 10 US adults meet diagnostic criteria for alcohol use disorder [1], and 25% engage in binge drinking (4+ drinks for women and 5+ for men in 2 hours) [2]. In addition, 3 in 10 adults use other drugs, like cannabis, stimulants, and opioids [2], and 4% meet diagnostic criteria for a drug use disorder [3]. Together, alcohol and other drug use (ie, substance use) are major public health burdens [4,5] and cost the United States $500 billion annually [6,7]. Only 10% of those with substance use disorder (SUD) seek specialty treatment, and of those who do not seek SUD treatment, 95% do not perceive a treatment need [8]. The reach of public health approaches to boost clinical screening for substance use problems [9] may be enhanced by developing and testing novel, technology-based strategies to identify individuals who use substances, including but not limited to those engaging in harmful use [10]. If effective at identifying such individuals, technology-based strategies could then be paired with alternative and innovative interventions, which may be scaled up and deployed to help individuals with milder SUD variants who may be less likely to seek formal care [11], as well as individuals who might benefit from harm reduction–based psychoeducation. One such technology-based strategy leverages data from commonly used social network sites (SNSs) such as Facebook, Twitter, and Instagram. More than 70% of US adults have at least one SNS account, including nearly 70% who use Facebook, 37% who use Instagram, and 22% who use Twitter [12]. Among young adults specifically, who are disproportionately represented among individuals with SUD and hazardous drinking [2], Instagram is nearly as popular as Facebook, with 67% versus 79%, respectively, having ever used these platforms [12].

As detailed below, emerging research suggests that individuals’ SNS posts can be leveraged to detect substance use [10,13,14]. However, these computational strategies function more efficiently and reliably with increasing amounts of data [15]. Thus, if certain types of individuals are less likely to participate or have lower levels of participation on certain SNSs, then it may be more difficult to detect substance use in these individuals, even if models are ostensibly well designed. As such, a greater understanding of the associations among individuals’ demographic characteristics, SNS participation, and substance use can help inform the utility of these computational screening tools. In the current study, we examined associations between substance use, including at-risk drinking and drug use, and Instagram posts and tested whether such associations differ as a function of age, gender, and race/ethnicity.

### SNS-Based Computational Strategies to Detect Substance Use Risk

In a prior study, our team [10] employed a novel deep neural network (ie, deep learning) framework [16] capable of processing data points with varying dimensions, including text and pictures of various sizes, to detect at-risk drinking and drug use from Instagram images, captions, and comments among a community sample of adults aged 18 and older, recruited primarily through Clickworker (Clickworker GmbH). Findings showed the deep learning model significantly predicted at-risk drinking, defined as having “had more than a few alcoholic drinks a day” at least once in the past year. In this model, the area under the receiver operating characteristic curve (AUROC) was 0.65, meaning there was a 65% chance that this novel classification model would correctly assign a higher score to a random positive example (participant reported at-risk drinking at least once in the past year) than a random negative example (participant reported no at-risk drinking in the past year). While the consensus is that AUROCs less than 0.7 provide low discrimination [17], the proof-of-concept study showed that a combination of visual and text-based SNS data could be used to detect self-reported at-risk drinking. The deep learning model was unable to detect other drug use any better than chance. Of note, the combination of images, captions, and comments evidenced superior detection of at-risk drinking compared with other combinations of Instagram content. To our knowledge, this study represents the only published research to date that has leveraged machine learning to detect individuals’ substance use from their SNS data in a nonclinical sample.

Studies showing that online forum content can be used to detect risk in clinical SUD samples are also instructive. Kornfield et al [13,14] used natural language processing in 2 studies, one of which also employed machine learning, to predict risk for negative substance use outcomes among individuals with both alcohol and other drug use disorders participating in a smartphone app online recovery forum. In the first study [14], they examined the utility of natural language processing with a Linguistic Inquiry and Word Count approach to predict binge drinking (5+ drinks for men and 4+ for women in 2 hours) in participants with alcohol use disorder. Individuals in this secondary analysis of a randomized controlled trial testing a smartphone-based intervention after residential treatment [18] were both randomized to receive the smartphone app intervention and participated in the application’s online forum between baseline (ie, residential treatment discharge) and the 4-month follow-up. Controlling for individual and system use characteristics (eg, number of messages posted), a greater percentage of words capturing swearing, negative affect, inhibition/control, and love and a lower percentage of words capturing higher-order cognitive processes (eg, insight) and achievement predicted past-month binge drinking at the 12-month follow-up [14]. In a related study including both participants from Gustafson et al [18] and participants with a range of SUDs recruited from primary care in Quanbeck et al [19], Kornfield et al [13] showed that decision tree machine learning algorithms can be used to detect recovery problems from online forum content. Algorithms included both Linguistic Inquiry and Word Count as well as Bag of Words natural language processing approaches. Recovery problems were determined by the research team based on a codebook informed by forum moderators’ perspectives regarding which posts warranted concern or intervention.

It is worth acknowledging the large and growing body of literature on the use of aggregated SNS big data to help surveil public health trends in drug use [20,21]. This big data surveillance literature and the use of SNS data to detect substance use risk share the use of SNS data as a proxy or marker of human behavior, which can then be downloaded for analysis. Aims of the current study, however, are intended to inform strategies that detect substance use at the individual level. Such a risk detection approach, when determined to be sufficiently reliable, would ultimately then be paired with interventions as mentioned above. When using SNS data in the context of drug use surveillance, data are analyzed in aggregate to identify macro-level trends and are thus outside the scope of this study of individual behaviors.

### Associations Between SNS Participation and Substance Use

Following from studies of computational methods using SNS data to detect substance use, it is also important to examine whether, and in what contexts, individuals who use substances also engage with SNSs to inform the utility of these technology-based screening tools. In a meta-analysis of 17 studies targeting adolescents and young adults, Curtis et al [22] found that self-reported alcohol consumption, including both general consumption and measures of risky drinking, is moderately associated with self-reported and hand-coded SNS engagement, including both alcohol-related SNS posts as well as exposure to alcohol-related SNS posts of others (overall *r*=0.36). In a related meta-analysis of 7 studies [22] authors similarly found that alcohol-related problems, as measured, for example, by the Alcohol Use Disorders Identification Test [23], were also moderately associated with SNS engagement (overall *r*=0.37). Thus, younger individuals may provide enough SNS data for these computational strategies to detect at-risk drinking.

Most studies examining the association between SNS participation and substance use to date have focused on the association between Facebook engagement and drinking. In an exception, Instagram participation was negatively related to past-month days of cannabis use but positively related to alcohol use in emerging adult (ie, aged 18-29 years) Instagram users recruited through Amazon mTurk [24]. Given the literature’s focus on Facebook and drinking among youth, studies that include individuals across age groups and that examine the association between substance use, including both alcohol and drug use, and popular platforms other than Facebook, such as Instagram, may help build on this emerging scientific literature.

### Demographic Factors That Moderate SNS–Substance Use Relationships

Knowledge about whether demographic characteristics moderate associations between SNS participation and substance use might further improve research on computational detection strategies. If, for example, there was an association between at-risk drinking and SNS participation only for younger individuals, this might reduce the utility of an SNS data–based model in detecting at-risk drinking for older individuals. To date, however, there are no existing studies that examine whether demographic characteristics moderate the association between substance use and SNS participation in community (ie, nonclinical) samples.

In the absence of prior work that might inform whether demographic characteristics moderate SNS–substance use relationships in the current study, it is worth mentioning that, consistent with the general population [12], emerging adults (aged 18-29 years) in SUD treatment report greater SNS participation compared with middle- and older-aged adults [25]. Similarly, in a nationally representative sample of US adults who resolved a substance use problem, emerging adults were more likely than their older counterparts to have used online resources, including but not limited to SNS platforms, to address their substance use or enhance their SUD recovery [26]. Also, in this same nationally representative recovery sample, Hispanic race/ethnicity relative to White race was related to a greater likelihood of recovery-related use of online resources, though men and women reported similar rates of this online help-seeking behavior. While these data are based on treatment and recovery samples, whereas the current study focuses on substance use in nonclinical samples, they suggest that any observed relationships between substance use and SNS participation might differ by age and race/ethnicity.

### Summary and Current Study

Emerging technology-based strategies to detect substance use with SNS data hold promise as scalable health risk screening tools. These computational strategies, of course, can only be effective among individuals who participate on SNSs. Even among those who participate, these screening tools are more powerful and, therefore, more useful, with increasing amounts of data. Thus, the real-world utility of these tools can be informed by research examining the associations between substance use, SNS participation, and demographic factors that moderate these relationships. Existing research suggests that substance use, including but not limited to hazardous use, is associated with greater SNS participation, but studies have focused primarily on drinking among young people on Facebook. To expand on this work, the current study targeted Instagram participation among adults of all ages and had the following aims: (1) to examine whether at-risk drinking and drug use is related to Instagram posts and (2) to examine whether these relationships between substance use and Instagram posts are moderated by age, gender, and race/ethnicity. We hypothesized that at-risk drinking would be related to more Instagram posts; we made no a priori hypotheses about drug use. We also hypothesized that the relationship between at-risk drinking and Instagram posts would be greater among younger individuals; we made no a priori hypotheses about other potential moderating demographic characteristics.

## Methods

### Procedure

As detailed in Hassanpour et al [10], study participants were recruited in the winter of 2016, primarily through the Clickworker crowdsourcing platform, which compensates individuals directly for study participation. Participants were also recruited via word of mouth and SNS advertisements on social media. Each recruitment avenue directed participants to the study website, where, following online consent, they completed an online survey consisting of demographic information and the National Institute on Drug Abuse’s (NIDA’s) Quick Screen substance use screener (see the “Measures” section below for more details of the NIDA Quick Screen) [27].

Instagram permitted the use of their application program interface (API) to collect participants’ data with individuals’ permission. Specifically, upon completion of the survey, the online study site linked participants to the Instagram gateway where they could give their permission to allow an application developed by our team to communicate with the Instagram API. If participants granted permission, their posts were downloaded onto a secure server and stored under an anonymized unique identifier for restricted use in this study. The team piloted the procedure and application, facilitating download from Instagram’s API, on 81 individuals, whose data were not included in the final, analyzed sample.

### Measures

#### Demographic Characteristics

Participants indicated their gender (male, female, transgender, or other), age in years, and race/ethnicity (Asian, Black, Hispanic/Latino, Native American/Alaskan Native, Native Hawaiian/Pacific Islander, White). We categorized age into groups of 18 to 25 years, 26 to 38 years, and 39 years or older, with cut-off points approximating 1 SD above and below the mean, while accounting for the theoretically important life stage of “emerging adulthood,” sometimes operationalized as ages 18 to 25 [28].

#### Instagram Posts

Instagram is a photo- and video-based SNS, accessible traditionally by smartphone app but also accessible with limited features via the website www.instagram.com. All participants had an Instagram account, though a subset had no content in their account. Participants were dichotomized into any versus no Instagram photo posts. For those with any posts, we included a count variable measuring the total number of posts. Video posts were excluded, as this content could not be analyzed with machine learning architecture used in the current study (see Hassanpour et al [10] for a detailed description of the machine learning approach).

#### Substance Use

Adapted from the NIDA Quick Screen [27], individuals reported frequency in the past year—never, once or twice, monthly, weekly, and daily or almost daily—of having “had more than a few alcoholic drinks a day,” which we refer to as at-risk drinking, using illegal drugs (ie, cannabis, stimulants, opioids, etc), which we refer to here simply as drug use, and using prescription drugs for nonmedical reasons (ie, opioid painkillers, benzodiazepines, and stimulants for attention-deficit/hyperactivity disorder), which we refer to here simply as prescription drug use. Consistent with NIDA’s guidance [27] and our past work [10], individuals who indicated “once or twice” or more frequently for each substance use category were counted as “positive” in screening for at-risk drinking, drug use, and prescription drug use. Of note, the NIDA Quick Screen assesses alcohol use with a criterion of 4+ drinks and 5+ drinks in 1 day for women and men, respectively. In the original study from which these data were derived [10], the research team decided to use having “had more than a few alcoholic drinks a day” instead of the NIDA criterion to reduce recall burden in the context of a very brief questionnaire. Also, while the NIDA Quick Screen includes an item for tobacco products, the current study focused on alcohol and drug use only.

### Participants

Out of a total of 3117 participants, 861 (27.62%) had an account with 0 posts and 2256 (72.37%) had an account with one or more posts. For those with one or more posts, the average number of posts was 201.9 (SD 394.7), ranging from 1 to 4999 posts. For substance use, in the past year, 55.34% (1725/3117) engaged in at-risk drinking, 22.30% (695/3117) used drugs, and 15.27% (476/3117) used prescription drugs.

With respect to demographic characteristics, participants were aged 29.9 years on average (SD 9.86; range 18-73 years), with 40.52% (1263/3117) aged between 18 and 25 years, 42.38% (1321/3117) between 26 and 38 years, and 17.10% (533/3117) 39 years and older. For gender, 37.12% (1157/3117) identified as male, 62.14% (1937/3117) as female, 0.38% (12/3117) as transgender, and 0.35% (11/3117) as other gender. For race/ethnicity, 6.48% (202/3117) identified as Asian, 16.27% (507/3117) as Black, 10.27% (320/3117) as Hispanic/Latino, 62.59% (1951/3117) as White, and 4.40% (137/3117) as other (including Alaskan Native/Native American or Hawaiian Native/Pacific Islander).

### Analysis Plan

For aim 1, we used logistic regression to examine the main effects of demographic characteristics and substance use variables on having an account with 0 versus any Instagram posts. We used negative binomial regression to examine the main effects of demographic and substance use variables on the number of Instagram posts for individuals. Since Instagram posts are count data and its distribution is overdispersed, we considered both Poisson regression, which assumes that the variance and mean are equal in the dependent variable, and negative binomial regression. Upon examination of the quantile-quantile plot (Multimedia Appendix 1), the distribution of Instagram posts was similar to a negative binomial distribution, supporting the use of a negative binomial regression to examine those factors associated with the number of Instagram posts [29].

For aim 2, we used the same logistic regression and negative binomial regression models as in aim 1 but examined the interaction effects between each of the demographic characteristics and at-risk drinking, drug use, and prescription drug use in the prediction of Instagram posts.

Of note, negative binomial regression models can yield inflated observed significance levels when there are high numbers of 0-count variables. In addition, the negative binomial regression models were unable to discriminate “true zeros” (ie, individuals who had Instagram accounts but 0 posts) from “artificial zeros” (ie, individuals who had 0 posts because they created accounts to participate in the study). In order to test whether removing those with 0 Instagram posts from the model would alter the pattern of findings, we conducted sensitivity analyses with zero-truncated negative binomial models, as in aims 1 and 2, with only individuals who had accounts with one or more Instagram posts (n=2256). The pattern of results for these sensitivity analyses was nearly identical, with the same significance testing results for all effects. Thus, we present only the primary analyses but include these tabulated sensitivity analysis results in Multimedia Appendix 2.

We used R 3.5.3 (The R Foundation) to conduct study analyses. All analyses tested significance at the .05 level. The institutional review board at Dartmouth College approved all study procedures. Data collection for this research project was conducted with informed consent from all participants and complied with the World Medical Association Declaration of Helsinki on Ethical Principles for Medical Research Involving Human Subjects.

## Results

### Associations Between Substance Use and Instagram Posts

#### Alcohol

At-risk drinking was significantly associated with a greater likelihood of any posts (Table 1) and greater number of posts (Table 2) compared with no at-risk drinking. Controlling for effects of demographic characteristics as well as drug use and prescription drug use, at-risk drinking was uniquely associated with 51.6% greater likelihood of any posts and 88.1% more posts.

**Table 1:** Logistic regression examining the associations between demographic characteristics, substance use, and the interaction between demographic characteristics and substance use with the likelihood of any Instagram posts.

Explanatory variables		Odds ratio (95% CI)	*P* value
Intercept		0.861 (0.655-1.133)	.29
**Age (years)**			
	18-25	1.596 (1.205-2.114)	.001
	26-38	1.000	N/A^a^
	39+	0.56 (0.411-0.762)	<.001
**Gender^b^**			
	Male	1.000	N/A
	Female	2.677 (2.077-3.452)	<.001
**Race**			
	White	1.000	N/A
	Asian	0.933 (0.608-1.433)	.75
	Black	1.676 (1.209-2.323)	.002
	Hispanic/Latino	1.653 (1.074-2.543)	.02
	Other	1.525 (0.831-2.797)	.17
**At-risk drinking**			
	No	1.000	N/A
	Yes	1.516 (1.048-2.192)	.03
**Drug use**			
	No	1.000	N/A
	Yes	1.771 (1.089-2.881)	.02
**Prescription drug use**			
	No	1.00	N/A
	Yes	1.153 (0.676-1.968)	.60
**Interaction terms**			
	Age 26-38 × at-risk drinking	1.000	N/A
	Age 18-25 x at-risk drinking	1.292 (0.856-1.950)	.22
	Age 39+ × at-risk drinking	0.661 (0.418-1.046)	.08
	Age 26-38 × drug use	1.000	N/A
	Age 18-25 × drug use	1.238 (0.711-2.154)	.45
	Age 39+ × drug use	0.824 (0.411-1.655)	.59
	Age 26-38 × prescription drug use	1.000	N/A
	Age 18-25 × prescription drug use	0.733 (0.407-1.322)	.30
	Age 39+ × prescription drug use	0.613 (0.306-1.227)	.17
	Male × at-risk drinking	1.000	N/A
	Female × at-risk drinking	1.391 (0.966-2.004)	.08
	Male × drug use	1.000	N/A
	Female × drug use	0.823 (0.497-1.364)	.45
	Male × prescription drug use	1.000	N/A
	Female × prescription drug use	0.627 (0.369-1.065)	.08
	White × at-risk drinking	1.000	N/A
	Asian × at-risk drinking	1.029 (0.471-2.25)	.94
	Black × at-risk drinking	0.709 (0.431-1.166)	.18
	Hispanic/Latino × at-risk drinking	0.992 (0.532-1.848)	.98
	Other × at-risk drinking	0.428 (0.17-1.076)	.07
	White × drug use	1.000	N/A
	Asian × drug use	2.127 (0.536-8.431)	.28
	Black × drug use	0.779 (0.404-1.503)	.46
	Hispanic/Latino × drug use	0.732 (0.321-1.668)	.46
	Other × drug use	2.268 (0.58-8.87)	.24
	White × prescription drug use	1.000	N/A
	Asian × prescription drug use	1.531 (0.464-5.05)	.49
	Black × prescription drug use	1.072 (0.527-2.182)	.85
	Hispanic/Latino × prescription drug use	0.745 (0.322-1.727)	.49
	Other × prescription drug use	1.46 (0.429-4.97)	.55

^a^N/A: not applicable.

^b^Although participants could report nonbinary gender, logistic regression models excluded other gender and transgender due to small cell sizes.

**Table 2:** Negative binomial regression examining the associations between demographic characteristics, substance use, and the interaction between demographic characteristics and substance use with the number of Instagram posts.

Explanatory variables		Probability ratio (95% CI)	*P* value
Intercept		55.339 (42.202-72.566)	<.001
**Age (years)**			
	26-38	1.000	N/A^a^
	18-25	0.911 (0.702-1.181)	.48
	39+	0.45 (0.331-0.612)	<.001
**Gender^b^**			
	Male	1.000	N/A
	Female	2.967 (2.326-3.785)	<.001
**Race**			
	White	1.000	N/A
	Asian	0.917 (0.602-1.395)	.68
	Black	0.905 (0.67-1.221)	.51
	Hispanic/Latino	2.005 (1.353-2.969)	<.001
	Other	0.822 (0.481-1.404)	.47
**At-risk drinking**			
	No	1.000	N/A
	Yes	1.881 (1.316-2.688)	<.001
**Drug use**			
	No	1.000	N/A
	Yes	1.289 (0.832-1.997)	.26
**Prescription drug use**			
	No	1.000	N/A
	Yes	0.915 (0.559-1.499)	.72
**Interaction terms**			
	Age 26-38 × at-risk drinking	1.000	N/A
	Age 18-25 × at-risk drinking	0.81 (0.571-1.148)	.24
	Age 39+ × at-risk drinking	0.802 (0.513-1.253)	.33
	Age 26-38 × drug use	1.000	N/A
	Age 18-25 × drug use	0.959 (0.624-1.476)	.85
	Age 39+ × drug use	1.401 (0.716-2.741)	.33
	Age 26-38 × prescription drug use	1.000	N/A
	Age 18-25 × prescription drug use	0.775 (0.474-1.269)	.31
	Age 39+ × prescription drug use	0.242 (0.123-0.476)	<.001
	Male × at-risk drinking	1.000	N/A
	Female × at-risk drinking	0.82 (0.589-1.143)	.24
	Male × drug use	1.000	N/A
	Female × drug use	1.073 (0.712-1.618)	.74
	Male × prescription drug use	1.000	N/A
	Female × prescription drug use	1.1 (0.693-1.747)	.69
	White × at-risk drinking	1.000	N/A
	Asian × at-risk drinking	1.246 (0.634-2.449)	.52
	Black × at-risk drinking	1.224 (0.794-1.888)	.36
	Hispanic/Latino × at-risk drinking	0.479 (0.284-0.81)	.006
	Other × at-risk drinking	1.211 (0.556-2.637)	.63
	White × drug use	1.000	N/A
	Asian × drug use	0.749 (0.3-1.873)	.54
	Black × drug use	1.048 (0.609-1.803)	.87
	Hispanic/Latino × drug use	1.375 (0.712-2.655)	.34
	Other × drug use	0.835 (0.333-2.093)	.70
	White × prescription drug use	1.000	N/A
	Asian × prescription drug use	2.076 (0.795-5.421)	.14
	Black × prescription drug use	0.738 (0.395-1.377)	.34
	Hispanic/Latino × prescription drug use	0.753 (0.361-1.574)	.45
	Other × prescription drug use	1.814 (0.679-4.847)	.24

^a^N/A: not applicable.

^b^Although participants could report nonbinary gender, negative binomial models excluded other gender and transgender due to small cell sizes.

#### Drug Use

Drug use was significantly associated with a greater likelihood of any posts (Table 1) but a similar number of posts (Table 2) compared with no drug use. Controlling for demographic characteristics and other substance use measures, drug use was uniquely associated with a 77% greater likelihood of any posts.

#### Prescription Drug Use

Prescription drug use was not associated with the likelihood of any posts (Table 1) or number of posts (Table 2).

### Moderation of the Instagram–Substance Use Associations by Demographics

#### Age

The association between prescription drug use and number of posts was moderated by age. For those aged 39 years or older, there was a significantly more negative association between prescription drug use and number of posts than for those aged 26 to 38 years (Table 2). Specifically, for those aged 39 years or older, prescription drug use was associated with 77.9% fewer posts compared with no prescription drug use, a significant effect, but for those aged 26 to 38 years, the association between prescription drug use and number of posts was nonsignificant. Associations for both at-risk drinking and drug use were not significantly moderated by age, for either any or number of Instagram posts.

Of note, the main effects of age showed that being aged 18 to 25 years was associated with a significantly greater likelihood of any posts (Table 1) but a similar number of posts (Table 2) compared with those aged 26 to 38 years. Both being aged 18 to 25 years and 26 to 38 years, compared with being aged 39 years or older, were associated with a significantly greater likelihood of any posts (Table 1) and a greater number of posts (Table 2).

#### Gender

Gender did not moderate any of the associations between substance use and any posts (Table 1) or number of posts (Table 2). Of note, there was a main effect of gender, such that being female was significantly associated with a greater likelihood of any posts (Table 1) and greater number of posts (Table 2) compared with being male.

#### Race/Ethnicity

The association between at-risk drinking and number of posts was moderated by race/ethnicity. For those identifying as Hispanic/Latino, the association between at-risk drinking and number of posts was nonsignificant, but for those identifying as White, at-risk drinking was associated with 88.1% more posts, a significant effect. Of note, however, main effects of race/ethnicity showed that compared with White identification, Black identification was significantly associated with a greater likelihood of any posts (Table 1), while Hispanic/Latino identification was significantly associated with a greater likelihood of any posts (Table 1) and greater number of posts (Table 2). Asian identification and other racial/ethnic identification was associated with a similar likelihood of any posts (Table 1) and similar number of posts (Table 2) compared with White identification.

## Discussion

### Summary of Findings

A greater understanding of the associations between substance use and SNS participation, as well as the demographic characteristics that moderate these associations, may help inform the utility of SNS data–based substance use screening tools [10]. In the present study, we showed that past-year at-risk drinking is associated with a greater likelihood of any Instagram posts. As hypothesized, at-risk drinking was associated with a greater number of posts, too, except for those identifying as Hispanic/Latino. Contrary to hypotheses, the relationship between at-risk drinking and Instagram posts did not differ as a function of age. Drug use (eg, cannabis, cocaine, heroin, etc) was associated with a greater likelihood of any posts but a similar number of posts compared to no drug use. Relative to no prescription drug use, any prescription drug use (eg, nonmedical use of opioids, benzodiazepines, stimulants, etc) was associated with fewer posts only among those aged 39 years and older, but it was associated with a similar likelihood of any posts, more generally. We outline the implications of these findings for researchers developing and testing strategies that employ SNS data to detect substance use.

### Association Between At-Risk Drinking and Instagram Posts

Findings showed that at-risk drinking, defined here as having “had more than a few alcoholic drinks a day” at least once in the past year, is uniquely associated with 88% more Instagram posts. For example, for an individual with demographic characteristics mapping onto all reference groups—male, aged 26 to 38 years, White, and no at-risk substance use—the model predicts 55 Instagram posts, which then increases to a predicted 104 posts if at-risk drinking is reported. Our data, which included adults aged 18 to 73 years and focused on Instagram participation, add to the body of literature showing that more drinking, as well as drinking problems, are associated with Facebook engagement among youth [22]. While needing to be replicated in other community samples, individuals with at-risk drinking may provide more data than their non–at-risk drinking counterparts, which can be leveraged in computational models of SNS data–based substance use risk detection.

We used a liberal definition of at-risk drinking, as recommended by NIDA Quick Screen guidelines, resulting in 55.34% (1725/3117) of the sample meeting at-risk drinking criteria. More than 70% of those defined as at-risk drinkers reported at-risk drinking (“more than a few alcoholic drinks a day”) one or two times in the past year [10]. While we did not assess for overall health, it is unlikely that alcohol consumption at this level would cause substantial physical consequences or map onto clinically significant alcohol use disorder. Thus, given that greater SNS participation may reflect greater social capital [30,31], that is, the social resources people can bring to bear on navigating challenges and problem-solving, it is possible that greater social capital may be associated with an increased likelihood of at-risk drinking when defined more liberally [32], as was the case here. In another study of Instagram users, for example, greater Instagram participation was associated with a composite of overall drinking and at-risk drinking (4+ and 5+ drinks in one day for women and men, respectively) only for those with the highest levels of peer belongingness [24]. Individuals with alcohol use disorder, on the other hand, are more likely to have reduced social involvement relative to those without SUD, based on the diagnostic criteria (eg, continuing drinking despite giving up hobbies, occupational or educational consequences, interpersonal difficulties, physical and mental health–related harms, etc). We might hypothesize, therefore, that individuals with alcohol use disorder would produce fewer SNS posts, potentially reducing the sensitivity of SNS data–based computational models. This type of curvilinear, inverted U-shaped relationship between at-risk drinking and SNS posts is speculative and should be tested in future work.

There was no association between at-risk drinking and number of Instagram posts for Hispanic/Latino individuals, but such individuals did have twice as many Instagram posts, overall, relative to White individuals. As such, it seems unlikely that using an SNS data–based method to screen for substance use would be any more challenging in Hispanic/Latino individuals relative to other racial/ethnic groups.

### Association Between Drug Use and Instagram Posts

Consistent with the NIDA Quick Screen [27], we analyzed drug use (ie, nonprescription drug use), such as cannabis, heroin, and cocaine, separately from prescription drug use (ie, nonmedical use of prescription medications), such as opioid painkillers, benzodiazepines, and stimulants, with disparate findings. Although drug use was associated with a 77% greater likelihood of any posts and similar number of posts, prescription drug use was generally not associated with either the likelihood of any posts or number of posts. For individuals 39 years and older, compared with those aged 26 to 38 years, prescription drug use may be associated with a *lower* number of posts. Thus, computational models that use Instagram data may have fewer posts with which to work if specifically aiming to detect prescription drug use among middle- and older-aged adults.

There are few prior studies of the association between SNS participation and drug use to which the current findings can be compared and contextualized. Exceptions have focused on cannabis, given it is the most widely used drug apart from alcohol [2] and its recreational use is now legal in Canada [33] and several states in the United States [34]. Bergman et al [24], for example, found cannabis use was negatively related to Instagram participation in a community sample of emerging adults aged 18-29 years. As the NIDA Quick Screen [27] queries frequency of use aggregated across drug types, we could not ascertain the association between Instagram posts and specific types of drug use. Studies that examine the utility of technology-based screening tools for opioid use in the context of the opioid overdose crisis [35], for example, may be warranted.

### Sample Generalizability

Study findings derive largely from participants recruited via Clickworker, a crowdsourced pay-for-performance site. While observed associations between certain demographic characteristics and Instagram post behaviors are not surprising, they are worth special mention, given their similarity with epidemiological data derived from nationally representative surveys among US adults. Specifically, we found that individuals aged 18 to 25 years were more likely to have at least one post compared with those aged 26 to 38 years, who, in turn, were more likely to have a post than those 39 years and older. Similarly, Pew Research Center [12] reports that 67% of individuals aged 18 to 29 years have an Instagram account, while 47% and 23% of individuals aged 30 to 49 years and 50 to 64 years, respectively, have an account. We found women were twice as likely to have at least one Instagram post compared to men, while Pew reported 43% of women to have an Instagram account compared to 31% of men. Finally, we found that Black and Hispanic/Latino individuals had the highest rates of Instagram engagement compared with other races/ethnicities, while Pew reported that 40% of Black and 51% of Hispanic/Latino individuals had an Instagram account compared to only 33% of White individuals. Thus, crowdsourced pay-for-performance or microtask sites, such as Clickworker and Amazon mTurk, may be reliable ways to achieve demographically representative groups of Instagram participants.

### Limitations

The following methodological limitations may be used to contextualize the study’s findings. First, when collecting the data, we did not capture the dates of each Instagram post and, by association, we are unable to determine how long individuals had been using Instagram. Given that computational models using SNS data are generally targeting current substance use, we would ideally be able to examine the association between substance use risk and recent SNS posts but were unable to do so with the current study methods. Second, the reasons that individuals had Instagram accounts with 0 posts remain unclear. For example, it may be that they simply observe the accounts of others (eg, “lurkers”), or they may have created an account for the sole purpose of participating in the current study to obtain compensation. Our analytic approach including both logistic regression (ie, any posts) and negative binomial regression (ie, number of posts) helps minimize the potential for such behavior to impact our pattern of findings. That said, our ability to interpret the real-world implications of the Instagram post outcomes is somewhat limited without this context. Finally, there were a set of limitations related to our substance use assessment. As mentioned above, our decision to target any instances of at-risk alcohol, prescription, and other drug use in the past year was consistent with the NIDA Quick Screen [27] but nevertheless constitutes a highly sensitive approach to identifying risk. While the detection of any substance use may aid critical prevention initiatives, future studies may also disentangle the reach of SNSs, including but not limited to Instagram, in detecting any substance use from their reach in detecting more clearly harmful variants (eg, screening tools for alcohol and other drug use disorder). Such studies might also examine whether the ability of such an SNS-based tool to detect SUD is moderated by number of Instagram posts. In addition, the measure of alcohol consumption used here queried instances of having “had more than a few alcoholic drinks a day” rather than the 4+ or 5+ per day criterion used in the NIDA Quick Screen. The ramifications of this adaptation are unclear.

### Summary and Conclusion

Greater knowledge of the association between substance use and SNS participation may inform the development and application of technology-based screening tools. Our findings suggest individuals with at-risk drinking and nonprescription drug use (eg, cannabis, cocaine, heroin, etc) may demonstrate greater participation on Instagram, which could be helpful when developing SNS-based models to detect substance use. On the other hand, the utility of SNS-based models to detect prescription drug use overall, and particularly among middle- and older-age adults, may be more limited, given their lower levels of Instagram participation. As we used a liberal criterion for at-risk drinking and aggregated several drug classes into just two categories, future work might focus on individuals with clinically significant drinking, such as those with alcohol use disorder, and individuals with specific types of drug use (eg, cannabis and opioids). Machine learning technologies that leverage individuals’ SNS data to passively screen for substance use may ultimately help reduce the overall burden of SUD and other harmful forms of drinking and drug use. As more is learned about SNS behaviors among those who use substances, researchers may be better positioned to successfully design and interpret these innovative risk detection approaches.

## Appendix

Multimedia Appendix 1 The distribution of number of Instagram posts was similar to a negative binomial distribution, leading us to use negative binomial regression (vs. Poisson regression) when examining associations between demographic characteristics, substance use, and number of Instagram posts.

Multimedia Appendix 2 Sensitivity analysis: zero-truncated negative binomial model examining the associations between demographic characteristics, substance use, and the interaction between demographic characteristics and substance use with the number of Instagram posts.

## Abbreviations

API: application program interface
AUROC: area under the receiver operating characteristic curve
NIDA: National Institute on Drug Abuse
SNS: social network site
SUD: substance use disorder
