# Supplementary figures and images for: Associations Between Substance Use and Instagram Participation to Inform Social Network–Based Screening Models: Multimodal Cross-Sectional Study
Source: J Med Internet Res. 2020 Sep 16;22(9):e21916. doi: 10.2196/21916 (PMC7527914; doi:10.2196/21916)

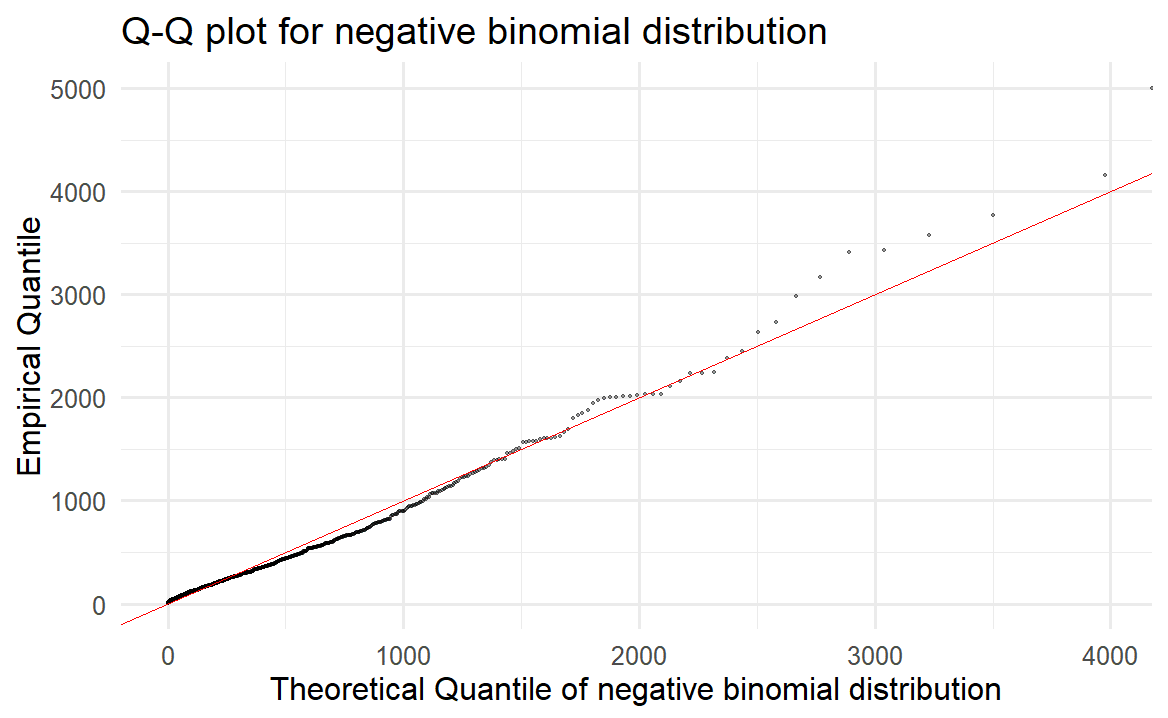

Supplement: Multimedia Appendix 1 [file jmir_v22i9e21916_app1.png]
