# Supplementary material for: Associations Between Substance Use and Instagram Participation to Inform Social Network–Based Screening Models: Multimodal Cross-Sectional Study
Source: J Med Internet Res. 2020 Sep 16;22(9):e21916. doi: 10.2196/21916 (PMC7527914; doi:10.2196/21916)
Supplement: Multimedia Appendix 2 [file jmir_v22i9e21916_app2.docx]

Supplemental Table 1

Sensitivity analysis: Zero-truncated Negative Binomial Model Examining the Associations Between Demographic Characteristics, Substance Use, and the Interaction between Demographic Characteristics and Substance Use with the Number of Instagram Posts

| Explanatory Variables | Probability Ratio | P-value |
| --- | --- | --- |
| (Intercept) | 98.362 (74.997, 129.007) | <.001 |
| **Age** |  |  |
| 26-38 |  |  |
| 18-25 | .801 (.63, 1.019) | .071 |
| 39+ | .544 (.398, .745) | <.001 |
| **Gender ^a^** |  |  |
| Male |  |  |
| Female | 2.163 (1.697, 2.756) | <.001 |
| **Race** |  |  |
| White |  |  |
| Asian | .908 (.601, 1.374) | .649 |
| Black | .752 (.571, .99) | .042 |
| Hispanic/Latino | 1.757 (1.223, 2.523) | .002 |
| Other | .715 (.434, 1.178) | .188 |
| **At-risk drinking** |  |  |
| No |  |  |
| Yes | 1.527 (1.081, 2.156) | .016 |
| **Drug use** |  |  |
| No | 1.000 | - |
| Yes | 1.022 (.679, 1.536) | .918 |
| **Prescription drug use** |  |  |
| No | 1.000 | - |
| Yes | .947 (.588, 1.525) | .822 |
| **Interaction terms**  Age 26-38 x At-risk drinking | 1.000 | - |
| Age 18-25 x At-risk drinking | .813 (.594, 1.111) | .194 |
| Age 39+ x At-risk drinking | .819 (.515, 1.302) | .398 |
| Age 26-38 x Drug use | 1.000 | - |
| Age 18-25 x Drug use | .971 (.656, 1.439) | .885 |
| Age 39+ x Drug use | 1.533 (.745, 3.153) | .245 |
| Age 26-38 x Prescription drug use | 1.000 | - |
| Age 18-25 x Prescription drug use | .768 (.487, 1.213) | .258 |
| Age 39+ x Prescription drug use | .316 (.144, .693) | .004 |
|  |  |  |
| Male x At-risk drinking | 1.000 | - |
| Female x At-risk drinking | .883 (.641, 1.215) | .444 |
| Male x Drug use | 1.000 | - |
| Female x Drug use | 1.266 (.865, 1.852) | .225 |
| Male x Prescription drug use | 1.000 | - |
| Female x Prescription drug use | 1.108 (.709, 1.73) | .653 |
|  |  |  |
| White x At-risk drinking | 1.000 | - |
| Asian x At-risk drinking | 1.149 (.602, 2.193) | .673 |
| Black x At-risk drinking | 1.385 (.944, 2.034) | .096 |
| Hispanic/Latino x At-risk drinking | .508 (.316, .816) | .005 |
| Other x At-risk drinking | 1.442 (.713, 2.915) | .309 |
| White x Drug use | 1.000 | - |
| Asian x Drug use | .745 (.329, 1.688) | .48 |
| Black x Drug use | 1.114 (.683, 1.817) | .666 |
| Hispanic/Latino x Drug use | 1.359 (.718, 2.574) | .346 |
| Other x Drug use | .775 (.361, 1.665) | .514 |
| White x Prescription drug use | 1.000 | - |
| Asian x Prescription drug use | 2.116 (.922, 4.857) | .077 |
| Black x Prescription drug use | .735 (.414, 1.304) | .293 |
| Hispanic/Latino x Prescription drug use | .856 (.396, 1.85) | .692 |
| Other x Prescription drug use | 1.682 (.685, 4.129) | .256 |
